# Ketamine’s Amelioration of Fear Extinction in Adolescent Male Mice Is Associated with the Activation of the Hippocampal Akt-mTOR-GluA1 Pathway

**DOI:** 10.3390/ph17060669

**Published:** 2024-05-22

**Authors:** Emilija Glavonic, Milorad Dragic, Milos Mitic, Minja Aleksic, Iva Lukic, Sanja Ivkovic, Miroslav Adzic

**Affiliations:** 1 Department of Molecular Biology and Endocrinology, “VINČA” Institute of Nuclear Sciences-National Institute of the Republic of Serbia, University of Belgrade, 11351 Belgrade, Serbia; emilija.glavonic@vin.bg.ac.rs (E.G.); milorad.dragic@bio.bg.ac.rs (M.D.); milos.mitic@vin.bg.ac.rs (M.M.); minjamil@vin.bg.ac.rs (M.A.); iva.lukic@vin.bg.ac.rs (I.L.); sivkovic@vin.bg.ac.rs (S.I.); 2 Laboratory for Neurobiology, Department of General Physiology and Biophysics, Faculty of Biology, University of Belgrade, 11158 Belgrade, Serbia

**Keywords:** fear-related disorders, fear extinction, ketamine, adolescence, mTOR signaling, hippocampus, prefrontal cortex

## Abstract

Fear-related disorders, including post-traumatic stress disorder (PTSD), and anxiety disorders are pervasive psychiatric conditions marked by persistent fear, stemming from its dysregulated acquisition and extinction. The primary treatment for these disorders, exposure therapy (ET), relies heavily on fear extinction (FE) principles. Adolescence, a vulnerable period for developing psychiatric disorders, is characterized by neurobiological changes in the fear circuitry, leading to impaired FE and increased susceptibility to relapse following ET. Ketamine, known for relieving anxiety and reducing PTSD symptoms, influences fear-related learning processes and synaptic plasticity across the fear circuitry. Our study aimed to investigate the effects of ketamine (10 mg/kg) on FE in adolescent male C57 BL/6 mice at the behavioral and molecular levels. We analyzed the protein and gene expression of synaptic plasticity markers in the hippocampus (HPC) and prefrontal cortex (PFC) and sought to identify neural correlates associated with ketamine’s effects on adolescent extinction learning. Ketamine ameliorated FE in the adolescent males, likely affecting the consolidation and/or recall of extinction memory. Ketamine also increased the Akt and mTOR activity and the GluA1 and GluN2A levels in the HPC and upregulated BDNF exon IV mRNA expression in the HPC and PFC of the fear-extinguished mice. Furthermore, ketamine increased the c-Fos expression in specific brain regions, including the ventral HPC (vHPC) and the left infralimbic ventromedial PFC (IL vmPFC). Providing a comprehensive exploration of ketamine’s mechanisms in adolescent FE, our study suggests that ketamine’s effects on FE in adolescent males are associated with the activation of hippocampal Akt-mTOR-GluA1 signaling, with the vHPC and the left IL vmPFC as the proposed neural correlates.

## 1. Introduction

Fear-related disorders, in particular post-traumatic stress disorder (PTSD), and anxiety disorders are highly prevalent and distressing psychiatric illnesses [1,2]. Characterized by excessive and persistent fear and anxiety, these conditions are systematically grouped together in the latest International Classification of Diseases (ICD-11). The behavioral perturbations observed with these disorders can primarily be attributed to the dysregulated acquisition and extinction of fear [3]. Additionally, one of the current first-line treatments for both PTSD and anxiety disorders—exposure therapy (ET)—relies heavily on the principles of fear extinction (FE) learning [4]. This underscores the significance of extinction paradigms in comprehending the pathophysiology of these disorders, as well as advancing the treatment options for both PTSD and anxiety disorders.

Adolescence is a period of increased susceptibility to the onset of a number of psychiatric illnesses [5], most notably anxiety [6,7]. Furthermore, approximately 75% of adults that experience fear-related disorders fulfill the diagnostic criteria during their late childhood and adolescence [8]. Adolescence is characterized by dynamic neurobiological changes across the fear circuitry, which includes the prefrontal cortex (PFC), hippocampus (HPC) and amygdala [9]. In particular, adolescence is marked by the delayed emergence of functional connectivity between the ventral HPC (vHPC) and the medial PFC (mPFC) [9], with significantly blunted prefrontal regulation of amygdala activity [10,11]. Adolescents demonstrate impaired FE compared to adults [12,13,14,15,16,17] and are more likely to relapse following ET [18,19]. In light of this, identifying pharmacological agents capable of enhancing FE in adolescents and ultimately improving their outcomes in ET represent a critical research imperative.

As a learning and memory paradigm, FE relies on glutamate transmission and activation of the signaling pathways involved in synaptic plasticity regulation within the fear circuitry. A number of studies have demonstrated the crucial role of hippocampal and cortical N-methyl D-aspartate (NMDA) and α-amino-3-hydroxy-5-methyl-4-isoxazole propionic acid (AMPA) receptors in extinction processes [13,20,21,22,23], in particular extinction consolidation [13,20,22]. Additionally, previous reports have highlighted brain-derived neurotrophic factor (BDNF) and Akt-mTOR signaling activity within these structures as potential mediators of extinction learning [24,25,26].

Ketamine, a non-competitive NMDA receptor antagonist, has recently emerged as a promising candidate for relieving anxiety [27,28,29] and significantly reducing the symptoms of PTSD [30,31,32]. Ketamine’s ability to promote the reprocessing of aversive experiences can be attributed to its influence on fear-related learning processes [33,34], as the drug has the capacity to promote structural and functional synaptic plasticity across the fear circuitry [35,36,37]. Ketamine preferentially blocks the NMDARs on GABAergic interneurons in the cortex and HPC [37,38], resulting in the disinhibition of presynaptic glutamatergic neurons, elevated glutamate release and increased activation of postsynaptic AMPA receptors [39,40,41]. Canonically, ketamine also upregulates BDNF expression and activates the mammalian target of rapamycin (mTOR) signaling cascade within these structures [39,42,43,44], thus affecting the molecular signals involved in fear extinction processes.

A number of studies in rodents have shown that ketamine facilitates FE in adults [25,45,46,47]. Ketamine’s effects on adult extinction learning are mediated by the mTOR signaling pathway in the mPFC [25] and can be associated with alterations in mPFC glutamatergic transmission [46]. On the other hand, only one study to date has reported on the behavioral effects of ketamine in a FE paradigm in adolescents [48]. Therefore, a comprehensive understanding of the underlying molecular mechanisms and the involvement of specific brain regions in ketamine’s effects on adolescent FE is currently lacking.

The main objective of our study was to examine ketamine’s impact on adolescent FE learning and analyze the molecular mechanisms linked to ketamine’s effects. The current body of evidence emphasizes the disinhibition hypothesis of ketamine’s mechanism of action in the PFC and HPC [37,49]. Therefore, by performing protein and gene expression analyses in these structures, we investigated the changes in glutamate signaling, the Akt-mTOR pathway and BDNF associated with ketamine’s effects on FE in adolescents. Finally, we conducted immunohistochemical analyses of the neuronal activity in different subregions of the HPC and PFC to define the neural correlates of ketamine’s actions in the context of adolescent FE.

## 2. Results

### 2.1. Ketamine’s Effects on the Locomotor Activity and Fear Extinction of Adolescent Mice

#### 2.1.1. Ketamine Does Not Affect the Locomotor Activity of Adolescent Mice

**Figure 1:**
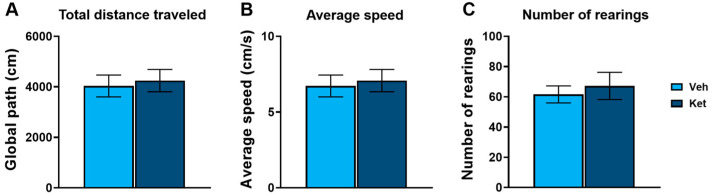
Open field test parameters in vehicle- and ketamine-treated male adolescent mice. (**A**) Total distance traveled as a measure of horizontal activity. (**B**) Average speed as a measure of horizontal activity. (**C**) Number of rearings as a measure of vertical activity. Data are presented as means ± SEM and were analyzed using independent samples *t*-tests. No statistically significant differences were found between vehicle (Veh) and ketamine (Ket) groups.

In this study, we first evaluated ketamine’s effects on the locomotion of adolescent mice using the open field test (OFT). We found no statistically significant differences in the total distance traveled (Figure 1A), average speed (Figure 1B) or number of rearings (Figure 1C) between the controls and the ketamine-treated mice.

#### 2.1.2. Ketamine Enhances Fear Extinction in Adolescent Males

Upon establishing that ketamine does not affect animals’ locomotion, we examined its influence on cued FE in adolescent male mice. We observed the animals’ freezing behavior and calculated various parameters related to the different stages of FE learning.

**Figure 2:**
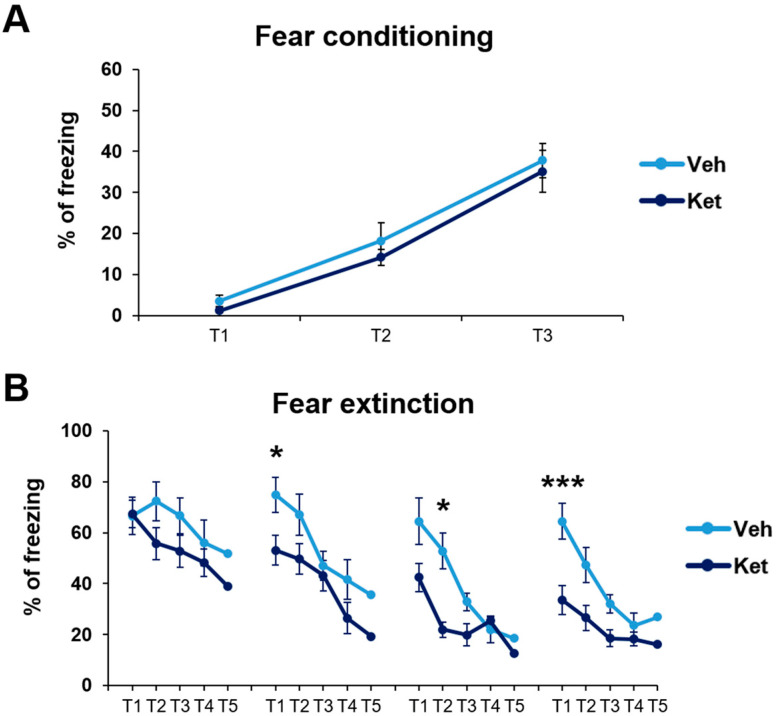
Cued fear conditioning and fear extinction paradigm in vehicle- and ketamine-treated male adolescent mice. (**A**) Adolescents’ freezing behavior across fear conditioning. (**B**) Adolescents’ freezing behavior across fear extinction. Data are presented as means ± SEM and were analyzed with two-way RM ANOVA, followed by post hoc Holm–Sidak correction, with conditioning/extinction trials as repeated variables and drug treatment as an independent variable. Statistically significant differences are given as *p* < 0.05. *; *p* < 0.001. *** Vehicle (Veh) vs. ketamine (Ket). T1–T5, Trials 1–5.

First, we established that fear was equally acquired in the treatment-naïve mice of both experimental groups. In this part of our research, we studied the animals’ freezing behavior during fear conditioning (FC). We found no statistically significant differences in fear acquisition across the three tone–foot shock presentations between the vehicle-designated and ketamine-designated animals (Figure 2A).

**Figure 3:**
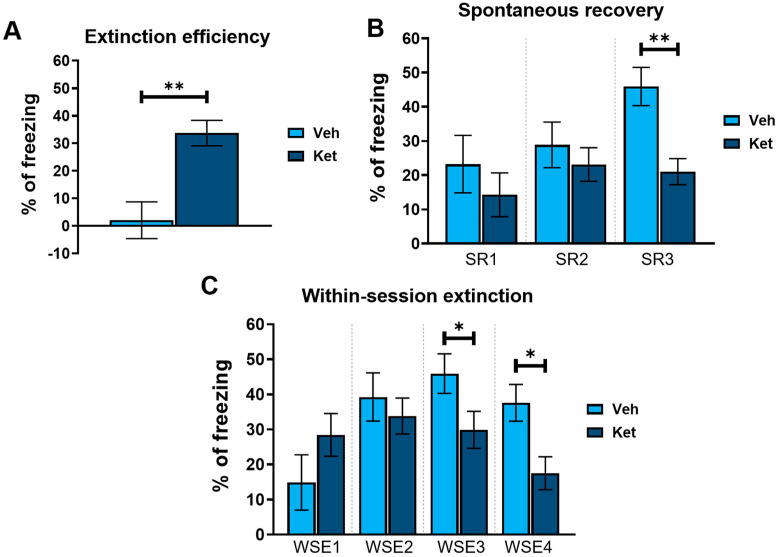
Different FE parameters in vehicle- and ketamine-treated male adolescent mice. (**A**) FE efficiency, calculated as follows: extinction efficiency  = E1T1 − E4T1 ([Ext Day 1, Trial 1] − [Ext Day 4, Trial 1]); (**B**) spontaneous recovery of fear, calculated as follows: SR1 = E2T1 − E1T5 ([Ext Day 2, Trial 1] − [Ext Day 1, Trial 5]); SR2 = E3T1 − E2T5 ([Ext Day 3, Trial 1] − [Ext Day 2, Trial 5]); SR3 = E4T1 − E3T5 ([Ext Day 4, Trial 1] − [Ext Day 3, Trial 5]); (**C**) within-session extinction, calculated as follows: WSE1 = E1T1 − E1T5 ([Ext Day 1, Trial 1] − [Ext Day 1, Trial 5]); WSE2 = E2T1−E2T5 ([Ext Day 2, Trial 1] − [Ext Day 2, Trial 5]); WSE3 = E3T1 − E3T5 ([Ext Day 3, Trial 1] − [Ext Day 3, Trial 5]); WSE4 = E4T1 − E4T5 ([Ext Day 4, Trial 1] − [Ext Day 4, Trial 5]). Data are presented as means ± SEM and were analyzed using independent samples *t*-tests for each extinction day across all behavioral parameters. Statistically significant differences are given as *p* < 0.05. *; *p* < 0.01. ** Vehicle (Veh) vs. ketamine (Ket). SR1–SR3, spontaneous recovery 1–3; WSE 1–4, within-session extinction 1–4.

Next, we explored how ketamine affects animals’ freezing behavior across 4 days of FE. Our results show that ketamine decreased the adolescents’ freezing during the first trial on Day 2 and Day 4 (Day 2, treatment: F_(1,30)_ = 5.03, *p* < 0.05; Day 4, treatment: F_(1,30)_ = 11.21, *p* < 0.05; Figure 2B), as well as during the second trial on Day 3 (Day 3, treatment: F_(1,30)_ = 5.69, *p* < 0.05; Figure 2B). Furthermore, to emphasize the difference in the animals’ long-term extinction success, as seen on Day 4, we introduced a parameter named “extinction efficiency”. It is calculated as the difference in the freezing percentages between the first trial of Day 1 and Day 4 of FE, thus reflecting the animals’ overall ability to extinguish fear responses. The ketamine-treated adolescents exhibited a significantly greater extinction efficiency compared to the control group (t_(30)_ = 3.90, *p* < 0.05; Figure 3A).

In addition, based on the freezing percentages outlined in Figure 2B, we calculated two behavioral parameters that further characterize long-term and short-term extinction learning. Animals’ ability to consolidate and retrieve extinction memory can be evaluated according to their spontaneous recovery of fear over time. Spontaneous recovery was calculated as the difference between the percentage of freezing at the end of the previous day and the percentage of freezing at the start of the next day of FE. We found that ketamine reduced the adolescents’ fear recovery compared to that of the controls, with statistically significant differences observed between the third and fourth day of FE (t_(30)_ = 3.69, *p* < 0.05; Figure 3B).

Finally, short-term extinction acquisition can be measured according to the within-session reduction in fear. Within-session extinction (WSE) is calculated as a differential between the freezing percentage at the beginning and the end of a single extinction session on any given day of FE training. The vehicle-treated adolescents demonstrated greater WSE compared to the ketamine group on both Day 3 and Day 4 of FE (WSE3: t_(30)_ = 2.08, *p* < 0.05; WSE4: t_(30)_ = 2.86, *p* < 0.05; Figure 3C). No statistically significant differences were found for the first two days of extinction.

### 2.2. Ketamine Upregulates Hippocampal Akt-mTOR-GluA1 Signaling in Fear-Extinguished Mice

After the behavioral analyses, we investigated the effects of ketamine on the expression of different glutamate receptors and kinases of the mTOR signaling pathway in the fear-extinguished mice. The levels of all the detected proteins were examined in synaptoneurosomes derived from the HPC and PFC of the adolescent males. Graph values of the protein levels within each tissue are presented as a % of the vehicle-treated controls, with the data given as means ± SEM.

**Figure 4:**
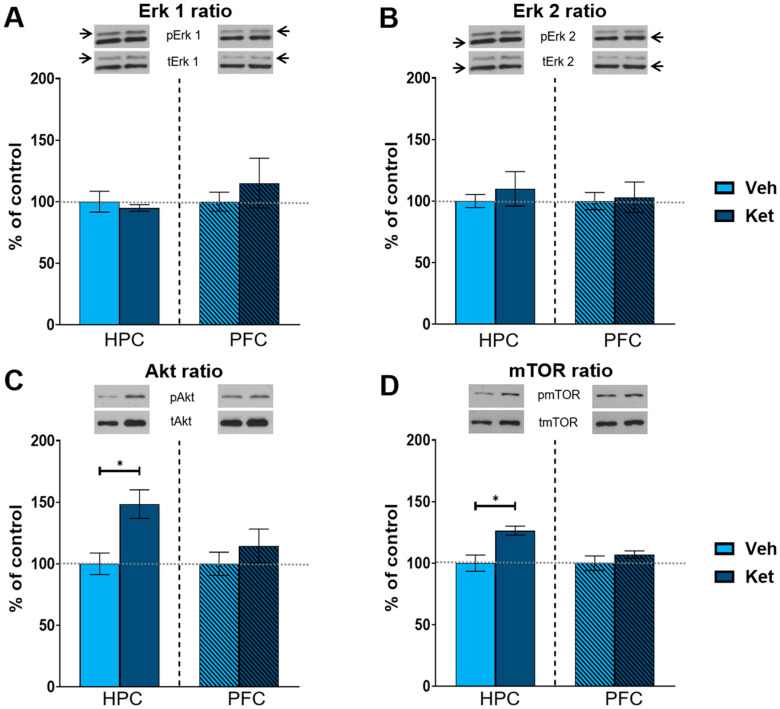
Protein expression of kinases of the mTOR signaling pathway. Levels of Erk1 (**A**), Erk2 (**B**), Akt (**C**) and mTOR (**D**) were detected in the synaptoneurosomal fractions derived from the hippocampus and prefrontal cortex of ketamine- and vehicle-treated adolescent mice. All proteins are represented as the ratio of their phosphorylated forms to their total forms. Graph values within each tissue are presented as a % of respective controls. Data are presented as means ± SEM and were analyzed using independent samples *t*-test within each tissue. Statistically significant differences are given as *p* < 0.05. * Vehicle (Veh) vs. ketamine (Ket). HPC, hippocampus; PFC, prefrontal cortex.

As kinase activity is best represented by the ratio of its phosphorylated to its total form, we focused on the ratio of the respective kinases of the mTOR pathway. Ketamine increased the hippocampal Akt and mTOR ratios compared to those of the controls (Akt ratio: t_(6)_ = 3.33, *p* < 0.05, Figure 4C; mTOR: t_(6)_ = 3.51, *p* < 0.05; Figure 4D and Figure S1), with no alterations detected in the PFC. However, we found no significant changes in the extracellular signal-regulated kinase 1 (Erk 1) and extracellular signal-regulated kinase 2 (Erk 2) ratios in either of the two examined tissues (Figure 4A,B and Figure S1).

**Figure 5:**
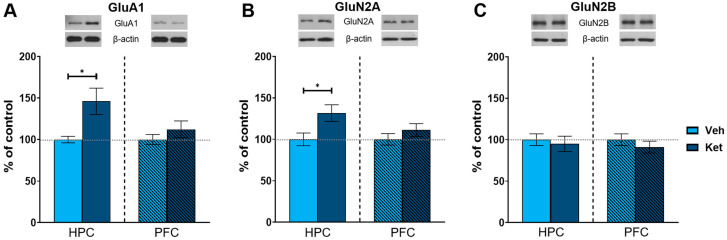
Protein expression of glutamate receptor subunits. Levels of GluA1 (**A**), GluN2A (**B**) and GluN2B (**C**) subunits were detected in the synaptoneurosomal fractions derived from the HPC and PFC of ketamine- and vehicle-treated adolescent mice. Graph values within each tissue are presented as a % of respective controls. Data are presented as means ± SEM and were analyzed with independent samples *t*-test within each tissue. Statistically significant differences are given as *p* < 0.05. * Vehicle (Veh) vs. ketamine (Ket). HPC, hippocampus; PFC, prefrontal cortex.

As an NMDA receptor antagonist, ketamine significantly affects glutamate signaling in the brain. Therefore, we next examined the effects of ketamine on the protein expression of key subunits of glutamate receptors in the fear-extinguished mice. The animals injected with ketamine exhibited increased levels of AMPA receptor subunit 1 (GluA1) and NMDA receptor subunit 2A (GluN2A) in their HPCs compared to the vehicle-treated mice (GluA1: t_(6)_ = 2.56, *p* < 0.05; Figure 5A; GluN2A: t_(6)_ = 2.50, *p* < 0.05; Figure 5B and Figure S1), with no differences in the PFC. We found no alterations in the protein expression of NMDA receptor subunit 2B (GluN2B) in either of the analyzed tissues (Figure 5C and Figure S1).

### 2.3. Ketamine Upregulates c-Fos Expression and Increases the Number of c-Fos+-Labeled Cells in the dHPC, vHPC and IL vmPFC of Fear-Extinguished Mice

To deepen our understanding of how ketamine enhances FE in adolescent mice, we further analyzed the expression of the neuronal activity marker, c-Fos, in various regions of the HPC and PFC. Specifically, we quantified the integrated fluorescence density derived from c-Fos+ cells and counted their number within the predefined regions of interest (ROIs), with the corresponding graph data presented as means ± SEM.

**Figure 6:**
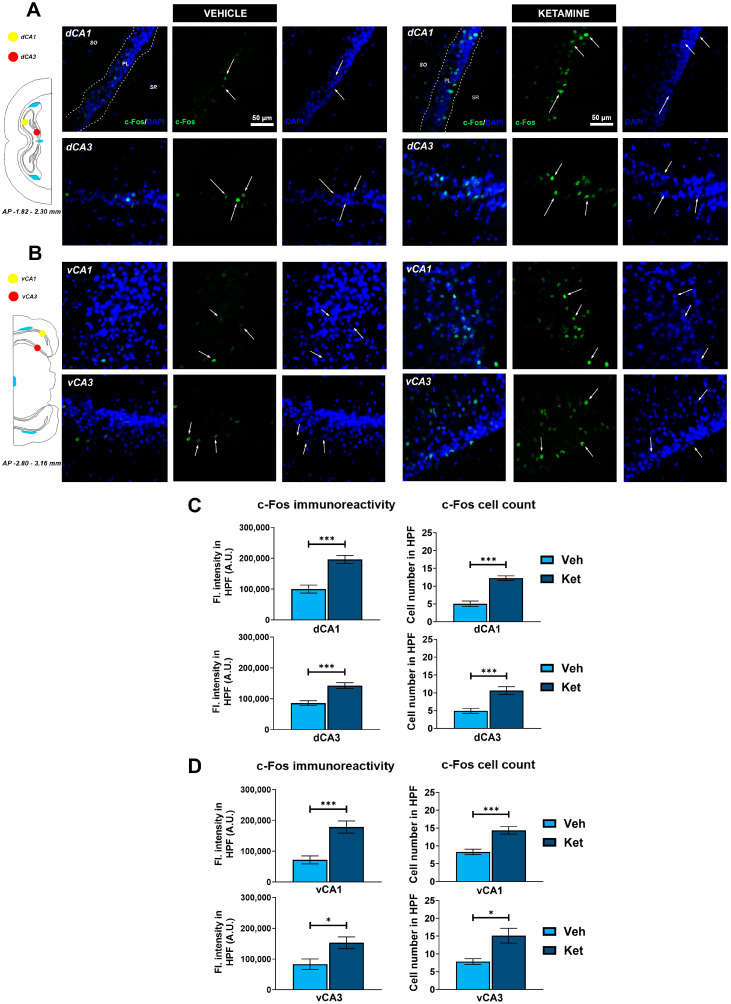
c-Fos expression in the dorsal and ventral hippocampus. Fluorescence staining of c-Fos (green) and DAPI (blue) in the CA1 and CA3 regions of the dorsal hippocampus (dHPC) and ventral hippocampus (vHPC) of vehicle-treated ((**A**,**B**), left panel) and ketamine-treated ((**A**,**B**), right panel) fear-extinguished adolescent mice. Separate c-Fos and DAPI micrographs were depicted as a confirmation of c-Fos colocalization with DAPI-stained cellular bodies (representative overlaps highlighted with white arrows). Representative graphs for c-Fos immunoreactivity ((**C**,**D**), left panel) and the number of c-Fos-labeled cells ((**C**,**D**), right panel) are presented as mean values ± SEM and were analyzed using independent samples *t*-test within each region. Statistically significant differences are given as *p* < 0.05. *; *p* < 0.001. *** Vehicle (Veh) vs. ketamine (Ket). dCA1, dorsal CA1 region; dCA3, dorsal CA3 region; vCA1, ventral CA1 region; vCA3, ventral CA3 region.

We examined the CA1 and CA3 regions of both the dorsal hippocampus (dHPC) and the vHPC (Figure 6A and Figure 6B, respectively). Since we found no inter-hemispheric ketamine-induced differences for any of the examined hippocampal subregions, all of the results are presented for the entire ROIs. We found that ketamine upregulated c-Fos expression in the fear-extinguished mice in the CA1 and CA3 regions of both the dHPC and the vHPC (dHPC CA1: t_(62)_ = 5.34, *p* < 0.05; Figure 6C; dHPC CA3: t_(29)_ = 4.61, *p* < 0.05; Figure 6C; vHPC CA1: t_(28)_ = 4.52, *p* < 0.05; Figure 6D; vHPC CA3: t_(14)_ = 2.72, *p* < 0.05; Figure 6D). Furthermore, the number of c-Fos+ cells within these regions was also greater in the mice receiving ketamine compared to that in the controls (dHPC CA1: t_(62)_ = 7.27, *p* < 0.05; Figure 6C; dHPC CA3: t_(30)_ = 4.39, *p* < 0.05; Figure 6C; vHPC CA1: t_(30)_ = 4.61, *p* < 0.05; Figure 6D; vHPC CA3: t_(14)_ = 3.25, *p* < 0.05; Figure 6D).

**Figure 7:**
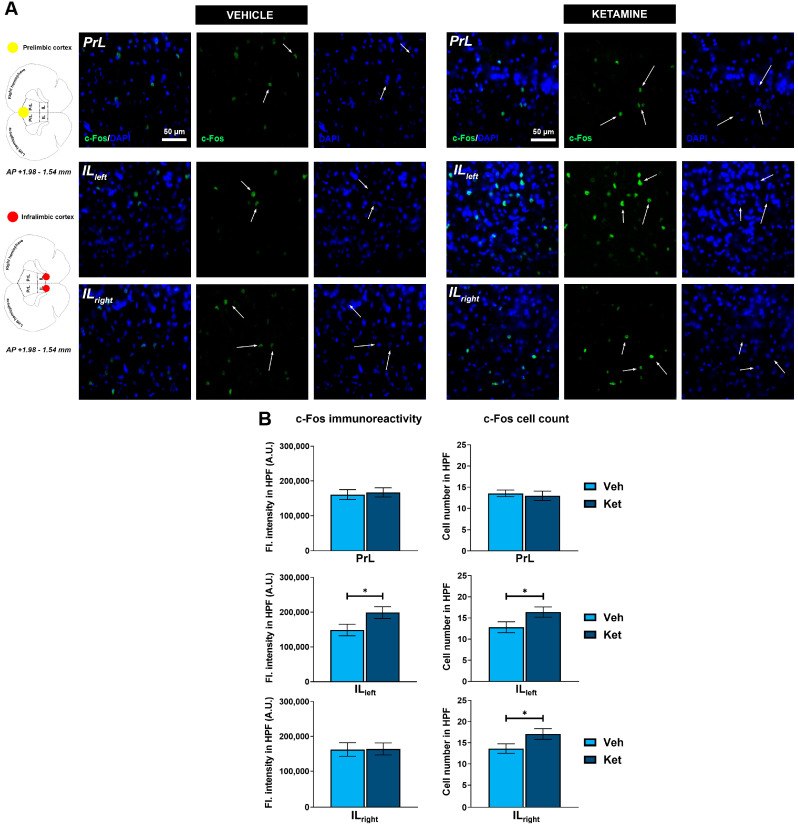
c-Fos expression in the ventromedial prefrontal cortex. Fluorescence staining of c-Fos (green) and DAPI (blue) in the prelimbic (PrL) and infralimbic (IL) areas of the ventromedial prefrontal cortex (vmPFC) of vehicle-treated ((**A**), left panel) and ketamine-treated ((**A**), right panel) fear-extinguished adolescent mice. Separate c-Fos and DAPI micrographs were depicted as a confirmation of c-Fos colocalization with DAPI-stained cellular bodies (representative overlaps highlighted with white arrows). Representative graphs for c-Fos immunoreactivity ((**B**), left panel) and the number of c-Fos-labeled cells ((**B**), right panel) are presented as mean values ± SEM and were analyzed using independent samples *t*-test within each region. Statistically significant differences are given as *p* < 0.05. * Vehicle (Veh) vs. ketamine (Ket). PrL, prelimbic cortex; IL, infralimbic cortex.

Regarding the PFC, we focused on the two ventromedially located regions crucial for fear memory processing, the prelimbic (PrL) and infralimbic (IL) cortices (Figure 7A). Within the PrL cortex, we found no differences in the expression of c-Fos between the control group and the ketamine group of fear-extinguished mice. However, ketamine boosted the c-Fos expression within the left hemisphere of the IL region (t_(30)_ = 2.12, *p* < 0.05; Figure 7B) while also increasing the number of c-Fos+ cells in both the left and right IL ventromedial PFC (IL vmPFC) (left IL: t_(30)_ = 2.10, *p* < 0.05; Figure 7B; right IL: t_(30)_ = 2.04, *p* = 0.05; Figure 7B).

### 2.4. Ketamine Upregulates BDNF Exon IV Expression in the HPC and PFC of Fear-Extinguished Mice

**Figure 8:**
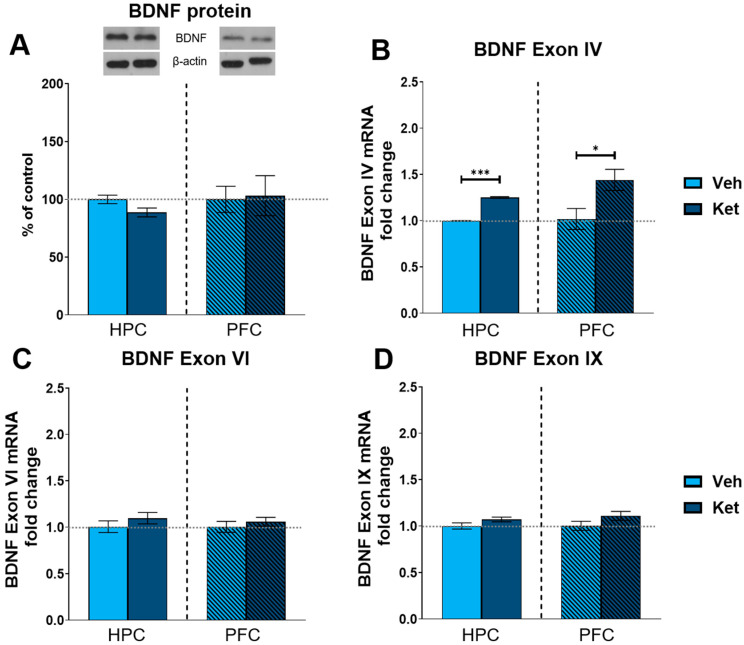
Protein and gene expression of BDNF and its exons. Protein levels of BDNF (**A**) were detected in the synaptoneurosomal fractions derived from the HPC and PFC of ketamine- and vehicle-treated adolescent mice. mRNA levels of BDNF exon IV (**B**), exon VI (**C**) and exon IX (**D**) were detected in the HPC and PFC of ketamine- and vehicle-treated adolescent mice. Graph values for protein expression within each tissue are presented as a % of respective controls. Graph values for mRNA expression within each tissue are presented as the fold change in respective controls. All data are presented as means ±  SEM and were analyzed using independent samples *t*-test within each tissue. Statistically significant differences are given as *p* < 0.05. * *p* < 0.001. *** Vehicle (Veh) vs. ketamine (Ket).

Finally, we explored ketamine’s influence on the protein expression of the most abundant synaptic plasticity marker in the brain, brain-derived neurotrophic factor (BDNF). We found no statistically significant differences in the BDNF levels between the vehicle- and ketamine-treated groups in either the HPC or the PFC of the fear-extinguished adolescents (Figure 8A and Figure S1). Additionally, we looked at the gene expression of several BDNF exons involved in long-term memory processing. Here, we found that ketamine upregulates the expression of BDNF exon IV in both the HPC and PFC following FE in adolescents (HPC: t_(6)_ = 26.8, *p* < 0.05; Figure 8B; PFC: t_(6)_ = 2.62, *p* < 0.05; Figure 8B), with no notable alterations observed for either exon VI or exon IX (Figure 8C and 8D, respectively).

## 3. Discussion

Ketamine has recently garnered much attention due to its rapid antidepressant effects [37,50], while numerous studies have demonstrated its effectiveness in reducing the symptom severity in anxiety disorders and PTSD [27,28,30,31,32]. Concurrently, several studies have shown that ketamine can positively impact fear-related learning processes [51,52], most notably FE [25,45,46,47], resulting in the suppression of learned fear responses.

Our previous work [13] demonstrated that male adolescents exhibit impaired extinction learning compared to adults. Here, we show that ketamine successfully ameliorates FE in male adolescents, which is linked with the activation of hippocampal Akt-mTOR-GluA1 signaling. Furthermore, we identify the vHPC and the left IL vmPFC as the likely neural correlates of ketamine’s effects on adolescent FE.

### 3.1. Ketamine Facilitates FE Consolidation/Retrieval in Adolescent Males

Considering that subanesthetic ketamine can have sedative properties, we aimed to ascertain whether 10 mg/kg of ketamine impairs animals’ locomotion when applied one hour before behavioral tests. The plasma half-life of i.p. administered ketamine in mice ranges from ~15 to 25 min [53,54], and 10 mg/kg of ketamine impaired locomotion in C57BL/6 mice 15 min after injection [55]. Nevertheless, consistent with Wei et al.’s study on adolescent males [48], we observed no differences in general locomotor activity between the ketamine-treated mice and their controls 60 min post-administration.

In order to conduct FE experiments, animals first need to be fear-conditioned. Here, it is crucial to differentiate between fear conditioning as utilized in our study and fear conditioning as a model of PTSD, which would require significantly stronger unconditioned stimuli to be applied (in terms of intensity, duration and the number of used stimuli [56]). In line with this, FE is considered an associative learning paradigm which serves as a valid model for the etiology and treatment of anxiety and fear-related disorders.

Alterations in extinction behavior can result from differences in fear acquisition. Therefore, we first demonstrated that both experimental groups acquired fear evenly across the three conditioning trials. As FC was carried out on drug-naïve animals, this was an expected outcome, which suggests that the effects of ketamine on FE result from the potentiation of extinction processes rather than higher levels of learned fear in the control group.

The adolescents treated with ketamine exhibited lower freezing percentages during the early extinction trials on Days 2–4, indicating ketamine’s potential to enhance FE. The ketamine group also showed a greater extinction efficiency and reduced spontaneous recovery of fear responses. Since both extinction efficiency and spontaneous fear recovery relate to long-term extinction learning [57], these results imply that ketamine affects the consolidation, retention and/or recall of extinction memory, processes known to be impaired in adolescent rodents [13,14,16]. In addition, ketamine attenuates fear recovery [45] and enhances extinction recall in adults [25], suggesting similar mechanisms of action across age groups. On the other hand, the vehicle-treated adolescents demonstrated more pronounced FE within two individual extinction sessions compared to the ketamine group. This result could indicate that ketamine negatively impacts extinction acquisition and short-term memory [57]. However, the greater within-session extinction of our controls likely stems from their higher initial levels of freezing on Days 3 and 4, which then enabled a more prominent reduction in fear within the individual extinction sessions.

After the initial extinction trials, both experimental groups reached comparable levels of freezing. A similar result was observed in adults [25], implying that ketamine has the greatest impact during initial exposure to cues previously associated with aversive stimuli. In recent years, preclinical data have also shown that administering ketamine before an acute stressor can attenuate learned fear and prevent the development of PTSD-like behavior in adults [51,58,59,60], highlighting prophylaxis as one of the potential approaches to ketamine’s clinical application. Here, our findings show that ketamine can reduce fear in adolescents by accelerating FE, suggesting that ketamine can serve as an adjunct to ET for this age cohort. While the preliminary data indicate that combining ET with ketamine for the treatment of fear-related disorders can improve the therapy outcomes in adults [61], further studies are needed to evaluate the efficacy of this approach in adolescents.

In general, the question of ketamine’s efficacy and subjects’ responsiveness to the drug is one of the main issues when it comes to its application in clinical settings. Regarding anxiety and PTSD, patients’ responsiveness to ketamine can vary between 67% [31,62] and 80% [30,63]. However, even with repeated ketamine infusions within clinical settings, participants in remission had a median time to relapse of 41 days [30], calling into question the durability of ketamine’s effects. It is also pertinent to highlight that the participants enrolled in these studies were diagnosed with treatment-resistant disorders, further emphasizing the importance of assessing ketamine’s therapeutic efficacy. In our study, we did not observe variability in the animals’ response to ketamine in terms of the treatment response. It is worth pointing out here that contrary to clinical trials, which rely on clearly defined criteria to determine the treatment response using validated scoring scales, preclinical FE studies lack such uniformly established criteria. Consequently, while they provide valuable insights into ketamine’s potential to improve therapy outcomes and offer an opportunity to investigate the underlying mechanisms, extrapolating the findings from preclinical FE studies to clinical settings regarding variability in the treatment response remains an exceedingly challenging task.

Another necessary area for future research includes the exploration of ketamine’s efficacy in improving FE in female cohorts. Based on numerous reports suggesting impaired FE in adolescent males [12,14,15,16,17] and our own prior findings, which revealed no differences in extinction learning between adolescent and adult females [13], we have conducted our experiments in adolescent males only. In general, the sex differences in FE between males and females can be attributed to the effects of estradiol on extinction learning, with females in the high-estrogen phases of their estrous and menstrual cycles exhibiting better FE [64,65,66]. Since females’ hormonal status is a potent FE modulator, it is a factor that should be considered in any extinction-based therapeutic interventions in the clinic. This is especially important since anxiety disorders in general manifest with a twice as high a prevalence in females compared to males [67], while females that experience trauma are also twice as likely to develop PTSD [68]. This emphasizes the critical need for efficient treatments targeting fear-related disorders within the female population, encouraging future studies of ketamine and FE in female cohorts.

### 3.2. Ketamine Upregulates the Hippocampal Akt-mTOR-GluA1 Pathway and c-Fos Expression in the Left IL vmPFC, dHPC and vHPC of Adolescent Fear-Extinguished Mice

Ketamine affects both functional and structural synaptic plasticity throughout the fear circuitry [35,36,37]. As an NMDA receptor antagonist, ketamine preferentially blocks the NMDARs on GABAergic interneurons in the cortex and HPC [37,38], consequently increasing fast AMPA-mediated glutamatergic transmission across these regions [39,40,41]. Additionally, ketamine upregulates BDNF expression and activates the mTOR signaling cascade [39,42,43,44], highlighting its multi-faceted impact on neuroplasticity processes. On the other hand, FE also engages similar synaptic plasticity mechanisms across the fear circuitry [26,69,70,71]; hence, ketamine likely potentiates extinction learning through converging molecular mechanisms in both the cortex and the HPC.

In our study, adolescents treated with ketamine had elevated levels of the GluA1 and GluN2A subunits in the HPC, suggesting that hippocampal GluA1 and GluN2A play a role in ketamine’s effects on adolescent FE. Previous reports indicate that ketamine increases GluA1 expression in the HPC [72], particularly in the vHPC [73], which may contribute to the ketamine-induced promotion of neurogenesis in this region [73]. Furthermore, Girgenti and colleagues observed that ketamine’s facilitation of FE in adults is at least partially dependent on AMPA receptor activity [25]. On the other hand, the involvement of the GluN2A subunit in ketamine’s effects on FE sparks an interesting discussion. While ketamine is a non-selective NMDA antagonist, it exhibits greater inhibitory potency at GluN2B-containing NMDARs [41,74]. At the same time, GluN2A activation is necessary for ketamine-induced enhancement of cortical activity [75] and the behavioral effects of low-dose ketamine [76], which aligns to an extent with our findings. However, further research is needed for a more comprehensive understanding of the involvement of specific NMDA receptor subunits in ketamine’s effects on FE learning.

**Figure 9:**
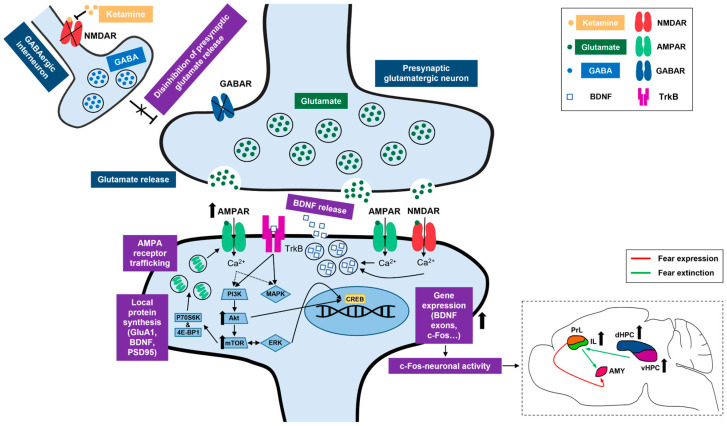
Summary of ketamine’s proposed mechanisms of action. Right bottom corner insert depicts a simplified view of ketamine’s proposed neural correlates in the context of adolescent fear extinction. NMDAR, N-methyl-D-aspartate receptor; GABA, gamma aminobutyric acid; GABAR, gamma aminobutyric acid receptor; AMPAR, alpha-amino-3-hydroxyl-5-methyl-4-isoxazolepropionic acid receptor; BDNF, brain-derived neurotrophic factor; TrkB, tropomyosin receptor kinase B; GluA1, AMPA receptor subunit 1; PSD95, postsynaptic density protein of 95 kDa; P70S6K, P70 S6 kinase; 4E-BP1, eukaryotic translation initiation factor 4E-binding protein 1; PI3K, phosphatidylinositol 3-kinase; Akt, protein kinase B; mTOR, mammalian target of rapamycin; MAPK, mitogen-activated protein kinase; ERK, extracellular signal-regulated kinase. CREB, cAMP response element-binding protein; c-Fos, an immediate early gene transcription factor; PrL, prelimbic cortex; IL, infralimbic cortex; AMY, amygdala; dHPC, dorsal hippocampus; vHPC, ventral hippocampus.

Ketamine also increased the Akt and mTOR activity in the HPC of fear-extinguished adolescents. Interestingly, the Erk 1 and Erk 2 activity remained unchanged, possibly due to the dosage, route and timing of ketamine application [33,77,78]. On the other hand, ketamine corrected abnormal fear memory processing in a mouse model of neurodevelopmental dysfunction [79] in an mTOR-dependent manner [80]. Additionally, ketamine’s ability to enhance extinction learning in adult rodents was linked to increased cortical activity of upstream mTOR kinases, including Akt. This behavioral effect was blocked by a selective mTOR inhibitor, rapamycin [25], indicating the functional significance of mTOR pathway in ketamine-assisted fear processing. It should also be noted that ketamine enhances mTOR phosphorylation in a time-course parallel to the elevation of synaptic GluA1 expression [72], one of the endpoints of mTOR signaling [81]. Consistent with this research, our results demonstrate a link between hippocampal Akt-mTOR-GluA1 signaling and ketamine’s effects on adolescent FE. A schematic representation of ketamine’s proposed mechanisms of action can be found in Figure 9.

Looking at the subregional level of HPC organization, the dHPC is mainly implicated in spatial and contextual learning, including context discrimination important for cued FE paradigms [82,83,84], whereas vHPC is more associated with stress and emotional memory processing [84]. We examined the c-Fos expression across these distinct HPC regions. c-Fos, a marker of neuronal activity [85], shows elevated expression levels following FE in regions of the fear circuitry associated with successful extinction learning [86,87]. In our study, ketamine induced significant upregulation of c-Fos expression in both the dHPC and vHPC, demonstrating its potential to modulate different facets of cued FE. Notably, prior studies have reported ketamine’s effects primarily within the vHPC [59,73,88]. On the other hand, Fraga and colleagues found that corticosterone significantly decreased the dendritic branching in both the dHPC and vHPC, an effect reversed by ketamine [89], supporting our findings of ketamine’s influence in both the dHPC and vHPC.

Regarding the PFC, we found no changes in the protein expression of any of the analyzed synaptic plasticity markers. However, we quantified the protein levels in the entire PFC and not solely in the vmPFC area, crucial to fear-related memory processing. Since the vmPFC constitutes only a small fraction of the overall PFC area, it is possible that the potential alterations in the plasticity-related proteins in the vmPFC were too subtle to be detected through Western blot analysis. Therefore, we conducted immunohistochemical analysis of the vmPFC regions, such as the PrL and IL areas, to discern their contribution to ketamine-assisted extinction learning in adolescents.

While the PrL cortex is associated with fear acquisition and the expression of conditioned fear responses [17,90], the IL cortex is more active during FE learning and extinction memory recall [17,86]. Ketamine increased the c-Fos expression in the left hemisphere of the IL cortex, a region previously highlighted to lack prototypical neural activity in adolescent mice during FE [17]. This finding holds further significance, as the left mPFC is known to play an inhibitory role in negative stress-related outcomes [91], including stress-induced anxiety [92]. Furthermore, transcranial magnetic stimulation of the left PFC region, functionally connected with the IL cortex in humans, resulted in reduced fear responses during extinction trials [93], which aligns with our observations that elevated activity within the left IL may underlie some of ketamine’s effects on adolescent FE. It should be noted that ketamine increased the number of c-Fos+ cells across the IL region in both hemispheres. This suggests that ketamine activates a comparable number of cells in both the left and right IL areas, with the intensity of neuronal activation notably stronger in the left hemisphere.

During adolescence, the fear circuitry undergoes substantial structural and functional changes, including an altered spine density and excitability across the mPFC favoring PrL activation [9,94] and delayed emergence of functional vHPC-mPFC connectivity [9], which contributes to increased responsiveness of the mPFC to amygdalar inputs [9]. Consequently, these neurodevelopmental changes may help explain adolescents’ inability to adequately extinguish learned fear responses. On the other hand, the activity in the vHPC-mPFC pathway may represent a neural substrate for some of ketamine’s antidepressant effects [95], with the IL mPFC in particular susceptible to the pharmacological actions of ketamine [96]. Here, we demonstrate that ketamine elevated the neuronal activity in the left IL vmPFC, as well as the dHPC and vHPC, of fear-extinguished mice. While acknowledging ketamine’s modulatory effects on the dHPC, the pivotal role of the vHPC in emotional memory processing and the significance of the vHPC-mPFC connection in adolescents’ FE suggest that the vHPC, alongside the IL vmPFC, may have a more prominent role in ketamine’s beneficial influence on adolescent FE. A simplified diagram depicting the proposed neural correlates of ketamine’s effects on adolescent FE is outlined in Figure 9.

### 3.3. Ketamine Upregulates BDNF Exon IV mRNA Expression in the HPC and PFC of Fear-Extinguished Mice

We found no discernible alterations in the BDNF protein levels between our experimental groups in either the HPC or the PFC. This was a surprising finding considering the role of BDNF in FE processes [26,69] and the well-established association between ketamine administration and elevated protein expression of BDNF [97,98,99,100]. However, given the alterations of exon-specific BDNF transcripts in fear-related learning processes [101,102,103], it is possible that ketamine affected the expression of distinct BDNF exons, such as exons IV and VI.

BDNF consists of multiple non-coding exons which are alternatively spliced onto the common coding exon (exon IX), responsible for encoding the precursor form of the BDNF protein [104]. It is hypothesized that these distinct exons differentially affect BDNF’s availability and intracellular targeting through the regulation of its translation and/or stability [105]. Exon IV transcripts are localized in the neuronal body and proximal dendrites, whereas exon VI transcripts have a distal dendritic localization [106]. Both somatic and dendritic BDNF splice variants regulate dendritic complexity [107]. Therefore, it is not surprising that both exons IV and VI are associated with long-term memory processing [108,109,110].

In our experiments, ketamine did not affect the mRNA expression of protein-coding exon IX in the HPC or the PFC of the fear-extinguished mice, consistent with our findings regarding BDNF protein expression. On the other hand, while we found no alterations in exon VI mRNA expression, ketamine increased the BDNF exon IV mRNA levels in both of the analyzed tissues, which aligns with reports of exon IV’s upregulation in response to ketamine [97,111]. Exon IV transcripts positively affect the number of primary, and to a lesser extent, secondary dendrites in primary cultures of hippocampal neurons [107], thus affecting synaptic reorganization. Furthermore, given the role of BDNF exon IV in the formation and maintenance of extinction memories [102,112], our results suggest that exon IV transcripts may contribute to ketamine’s positive effects on adolescent FE. This interpretation is further supported by the finding that the combination of ketamine and FE is associated with hypomethylation at the BDNF exon IV promoter and increased BDNF exon IV mRNA levels in the mPFC and HPC [45].

### 3.4. Limitations

Our research should be interpreted taking into account several limitations. Following our established FE protocols [13] and the experimental design of Wei and colleagues [48], ketamine was administered four times, once before each of the four extinction sessions, with molecular analyses conducted after the fourth day. This approach enabled us to identify molecular alterations associated with improved FE following our ketamine administration regimen. However, given some of the side effects associated with ketamine’s application [113], it would also be interesting to compare the efficacy and molecular signatures of repeated administration with a single dose of ketamine in the context of adolescent FE. While such an experimental framework was beyond the scope of our study, it is important for subsequent investigations to address this question and elucidate the optimal ketamine dose regimen for improving FE in adolescents.

In addition, as we analyzed a large number of proteins in the synaptoneurosomal cell fraction, we pooled our samples, which precluded us from correlating the protein levels with individual behavioral outcomes. For consistency, the same method was applied to our gene expression analyses. While this approach does effectively reflect intergroup differences, it is not suited to the characterization of individual variations in the underlying molecular mechanisms associated with ketamine’s influence on extinction learning, which is a research avenue worth exploring in the future.

## 4. Materials and Methods

### 4.1. Animals

All the experiments were conducted on adolescent male C57BL/6 mice (aged 29 days) acquired from the Jackson Laboratory and subsequently bred in our facilities. A total of 44 animals were used for the study. The mice were housed in standardized cages with ad libitum access to food and water, under a 12 h light/dark cycle in a temperature-controlled room set at 20 ± 2 °C. Animals from different litters were randomly assigned to each experimental group to account for litter-driven effects. All the experimental procedures were approved by the Ethical Committee of the “Vinča” Institute of Nuclear Sciences, National Institute of the Republic of Serbia (Application No. 02/2023), and by the Ethical Committee of the Veterinary Directorate of the Republic of Serbia (Approval No. 323–07–04156/2023–05). All the animal procedures complied with the EU Directive 2010/63/EU for animal experiments.

### 4.2. Drugs

Ketamine hydrochloride (100 mg/mL) was purchased from Richter Pharma and diluted to 1 mg/mL in 0.9% (*v*/*v*) saline. Ketamine was administered i.p. at a volume of 10 mL/kg and a dose of 10 mg/kg. This dose of ketamine was selected based on its previously demonstrated efficacy in improving FE in adolescent mice and was administered four times in total, following the protocol established by Wei et al. [48]. The vehicle-treated groups were injected with a mass-adjusted volume of saline.

### 4.3. Experimental Design

Upon reaching adolescence, that is, postnatal day 29 (P29), different animal cohorts were subjected to either locomotion tests in the open field arena or the cued FE protocols. Mice were randomly assigned to either the vehicle-treated or ketamine-treated groups for both sets of experiments.

**Figure 10:**
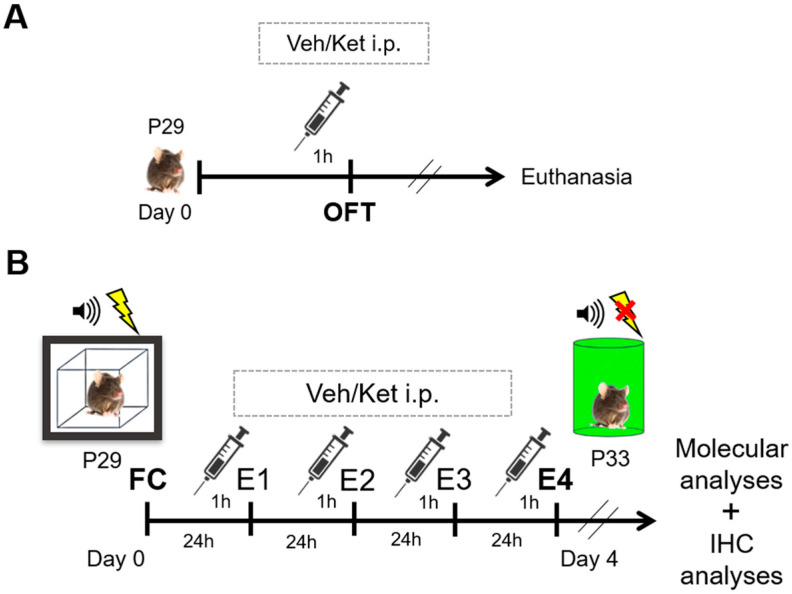
Schematic representation of experimental procedures. (**A**) Experimental protocol for the open field cohort. (**B**) Experimental protocol for the fear conditioning/extinction cohort. Abbreviations—P29: postnatal day 29; P33: postnatal day 33; OFT: open field test, Veh: vehicle; Ket: ketamine; FC: fear conditioning; E1–E4: FE days 1–4; IHC, immunohistochemistry.

Before assessing ketamine’s impact on FE, we evaluated its effects on mice locomotion in the OFT to account for any sedative properties that may have complicated the interpretation of the experimental results (Figure 10A). Mice were acutely injected with a mass-adjusted volume of saline or 10 mg/kg of ketamine one hour before the OFT to match the timing of the drug application in the FE experiments. Each of the two experimental groups comprised six animals that were not used for further analyses.

For the FE experiments, each group consisted of 16 fear-conditioned mice subsequently subjected to 4 consecutive days of extinction learning (Figure 10B). The animals were given a mass-adjusted volume of saline or 10 mg/kg of ketamine i.p. one hour before each extinction session across four days of extinction. To mitigate the effects of the environmental and handling variables, both the vehicle and ketamine group were subjected to identical conditions over the course of the experiment, including being handled by the same experimenter, as well as the injections and testing being conducted at consistent times of day. One hour after their last behavioral test, all the animals were euthanized by cervical dislocation, and their brain tissues were collected for further molecular analyses. Their whole brains were used for cryosectioning and the immunohistochemical analyses.

### 4.4. Behavioral Analyses

#### 4.4.1. Open Field Test

A 10 mg/kg dose of ketamine or a mass-adjusted volume of saline was administered i.p. 1 h before the OFT. In this paradigm, the animals (*n* = 6 per group) were placed into the corner of a square-shaped arena (52.5 × 52.5 × 30 cm), which was divided into nine equal squares for subsequent computer software analysis. The mice were left to explore the arena for 10 min, during which they were videotaped and their activity measured using the TSE VideoMot2 software (version 5.75; TSE Systems, Bad Homburg, Germany). We assessed ketamine’s influence on the mice’s locomotion by analyzing three parameters: total distance traveled and average speed for horizontal activity, as well as the number of rearings for vertical activity. The arena was cleaned with 70% ethanol solution after each animal.

#### 4.4.2. Cued Fear Conditioning and Extinction

Cued FC took place in a soundproof, specialized square chamber (Ugo Basile S.R.L., Gemonio, Italy), with foot shocks delivered via metal rods on the chamber floor. During a single conditioning trial, the mice (*n* = 16 per group) were first acclimated to the conditioning chamber and sprayed with 0.1% (vol) peppermint extract dissolved in 70% EtOH for 2 min. They were then fear-conditioned using three tone– shock pairings, where a 30 s (5 kHz, 70 dB) tone (conditioned stimulus, CS) co-occurred with a 0.7 mA foot shock (unconditioned stimulus, US) during the last second of its presentation. The CS-US presentations were separated by intervals of 30 s (intertrial intervals, ITIs). After the final stimuli pairing, the animals remained in the conditioning chamber for 1 min before being returned to their home cages. The arena was thoroughly cleaned with peppermint-scented ethanol between each mouse.

Following the FC, the mice received ketamine or the vehicle 1 h before the first of four extinction trials. All subsequent extinction sessions were conducted 24 h apart, with ketamine/the vehicle applied 1 h before each session. To eliminate the influence of the conditioning context on FE, all the extinction sessions were carried out in a novel arena (green cylindrical chamber scented with 0.1% (vol) lemon extract dissolved in 70% EtOH). The animals were given 2 min to adjust to the chamber conditions. They were then exposed to five 30 s tones (5 kHz, 70 dB) in the absence of US, with the ITIs between the tones lasting 30 s. Following the final tone presentation, the mice were left in the arena for 1 min before being returned to their home cages. Lemon-scented ethanol was used to clean the arena after each animal.

All the behavioral tests were conducted using the EthoVision XT behavioral software (version 11.0) designed by Noldus (Wageningen, the Netherlands), with the mice videotaped for later analysis of their freezing behavior by researchers blind to the experimental treatments. Freezing is defined as the lack of visible muscle movements except for the ones required for breathing [17] and was quantified as a reliable measure of fear in rodents. The percentage of time spent freezing during the CS presentation was calculated by dividing the number of seconds spent freezing by the duration of the stimulus (30 s). The FC and FE procedures were carried out according to protocols established by Pattwell and colleagues [17].

### 4.5. Preparation of the Crude Synaptosomal Fraction

The crude synaptosomal fractions were prepared from the previously dissected HPC and PFC tissues (*n* = 8 animals per group) per the protocol established by Ronald Duman’s lab [43,81]. In brief, tissue was homogenized by hand strokes in buffer containing 0.32 M sucrose, 20 mM HEPES (pH 7.3), 1 mM EDTA, 1 protease inhibitor cocktail, 5 mM NaF and 1 mM NaVO3 and subsequently centrifuged for 10 min at 2800 rpm. The pellets containing the nuclear fractions were discarded, while the supernatants were further centrifuged at 12,000 rpm for 10 min to obtain pellets of the crude synaptosomes. Finally, these pellets were resuspended and sonicated in RIPA lysis buffer containing 50 mM Tris–HCl (pH 7.5), 150 mM NaCl, 1% Triton X-100, 0.1% SDS, 2 mM EDTA, 1 mM NaVO3, 5 mM NaF and 1 protease inhibitor cocktail, which yielded the crude synaptosomal fractions used for the Western blot analyses.

### 4.6. Western Blot Analysis

The protein concentration was determined through a spectrophotometric method adjusted by Markwell [114]. According to Laemmli [115], the samples were then incubated in denaturing buffer at 100 °C for 5 min. A total of 40 μg of the proteins was separated using SDS-PAGE electrophoresis on 7.5%, 10% and 15% gels at a constant voltage (100 V). The proteins were then transferred using a blot system (Mini Trans-Blot, Bio-Rad, Hercules, CA, USA) onto a PVDF membrane (Immobilon-P membrane, Millipore, Burlington, MA, USA) for 2 h at 400 mA or overnight at a constant voltage (30 V). After the transfer, the membranes were blocked for 1 h in 5% non-fat dry milk dissolved in phosphate-buffered saline (PBS) and incubated in primary (overnight at 4 °C) and secondary HRP-conjugated antibodies (2 h at room temperature). The dilutions and catalog numbers of the primary antibodies are presented in Table 1, with β-actin applied as the loading control. The secondary antibodies used for detection were rabbit anti-mouse IgG-HRP and goat anti-rabbit IgG-HRP (Abcam, Cambridge, UK). The protein bands were detected using the Pierce™ enhanced chemiluminescent reagent and SuperSignal Femto Maximum Sensitivity Substrate (Thermo Scientific, Waltham, MA, USA) after X-ray film exposure (Fujifilm, Tokyo, Japan). The signals were analyzed according to densitometry using the ImageJ PC software (https://imagej.nih.gov/ij/; NIH, Bethesda, MD, USA), with the amounts of all proteins normalized to the β-actin levels.

### 4.7. Immunohistochemistry and Immunofluorescence Microscopy

For immunohistochemistry, the brains were carefully removed from the skull (*n* = 4 animals per group), fixed in 4% PFA for 24 h, cryoprotected in graded sucrose solution (10–30% in 0.2 M phosphate buffer) and frozen at −80 °C prior to their sectioning. Then, 20 µm thick coronal cryosections of the PFC, containing the PrL and IL regions, were dissected between 1.98 and 1.54 mm anterior to Bregma, whereas the dorsal dHPC and vHPC sections were taken between 1.82 and 3.16 mm posterior to Bregma. The cryosections were left to dry for several hours at room temperature (RT), with the microscopic slides then stored at −20 °C until further use.

Before staining, the slides were kept at RT for 30 min, rehydrated in PBS and incubated with 5% donkey serum at RT for 1 h to block non-specific antibody binding. Next, the sections were probed overnight with a c-Fos primary antibody (1:200 dilutions, Abcam, ab190289) at +4 °C in a humid chamber. After repeated washing steps in PBS, the sections were incubated with a fluorescent anti-rabbit secondary antibody (1:400 dilutions; Alexa Fluor 568, Invitrogen, Carlsbad, CA, USA) for 2 h at RT in a humid dark chamber. The sections were again washed in PBS and stained with 4′,6-diamidino-2-phenylindole (DAPI, Sigma-Aldrich, St. Louis, MO, USA) to visualize the cellular layers of the sectioned tissue. After the DAPI stain was washed off with PBS, the sections were mounted with Mowiol (Calbiochem, La Jolla, CA, USA) and analyzed using a confocal laser scanning microscope (LSM 510, Carl Zeiss GmbH, Jena, Germany), using a HeNe (543 nm) laser, a 40× DIC oil objective and a monochrome Axiocam ICm1 camera (Carl Zeiss GmbH, Oberkochen, Germany). DAPI was visualized using an epifluorescent lamp.

### 4.8. Image Quantification

For quantification of the c-Fos+ cells, images of the PrL and IL cortices, as well as the CA1 and CA3 regions of the dHPC and vHPC, were captured in the high-power field (HPF = 0.054 mm^2^, 1024 × 1024 pixels, 1 µm = 4.42 pixels) using a 40× objective. Depending on the size of the region, we captured one or two images per hemisphere per region from two sections per brain, with the first image taken next to the interhemispheric fissure and the second one taken deeper in the region. The total number of images for quantification was 32 per group for the PrL cortex, the IL cortex, and the dorsal CA1 region (dCA1), and 16 per group for the dorsal CA3 region (dCA3), the ventral CA1 region (vCA1) and the ventral CA3 region (vCA3). For quantification of the fluorescent intensity, raw multi-image IF micrographs were used to measure the integrated fluorescence density, expressed as arbitrary units (AUs), from the six predefined ROIs using the ImageJ PC software (https://imagej.nih.gov/ij/; NIH, Bethesda, MD, USA). Additionally, we performed a total c-Fos+ cell count from the examined ROIs.

### 4.9. RNA Extraction and Reverse Transcription

Total RNA from the animals’ HPCs and PFCs (*n* = 4 animals per group) was extracted with ROTIZOL Reagent (Carl Roth, Karlsruhe, Germany) in accordance with the manufacturer’s instructions. The tissue was weighed and homogenized in 1 mL of ROTIZOL Reagent per 100 mg of tissue in a Potter-Elvehjem Teflon–glass homogenizer. The tissue homogenates were incubated for 10 min at 30 °C, and 0.2 mL of chloroform (per 100 mg of tissue) was added. After vigorous shaking, the samples were incubated for 3 min at 30 °C and then centrifuged at 12,000× *g* for 15 min at 4 °C. The aqueous RNA-containing phase was mixed with 0.5 mL of isopropanol (per 100 mg of tissue), incubated for 10 min at 30 °C and centrifuged at 12,000× *g* for 10 min at 4 °C. The pellets containing the RNA were then resuspended in 75% ethanol, centrifuged at 7500× *g* for 5 min at 4 °C, air-dried and dissolved in 50 µL of 0.1% DEPC water. The obtained RNA was stored at −80 °C until further analysis. The RNA concentration was determined by measuring the absorbance at 260 nm using a NanoDrop spectrophotometer, and its purity was assessed by calculating the ratio between the absorbance levels at 260 and 280 nm. The RNA integrity was determined through visualization of the 28S and 18S rRNA bands, separated on 1.5% agarose gel previously loaded with 2 mg of RNA. For cDNA synthesis, a High-Capacity cDNA Reverse Transcription Kit (Applied Biosystems, Waltham, MA, USA) was used according to the manufacturer’s instructions, with the cDNA subsequently kept at −20 °C until further use.

### 4.10. qRT-PCR

The studied genes were amplified in a 7500 Real-Time PCR System (Applied Biosystems) with Power SYBR Green PCR Master Mix (Applied Biosystems), in line with the manufacturer’s instructions. The mRNA levels were detected for BDNF exon IV, BDNF exon VI and BDNF exon IX, while the housekeeping gene hypoxanthine phosphoribosyltransferase 1 (HPRT) was used as an endogenous control to normalize the amount of total mRNA in each sample. The specific primer sequences are listed in Table 2. Amplification of target cDNAs was performed under the following conditions: hold at 95 °C/10 min, 40 cycles of denaturation at 95 °C/15 s, annealing and extension at 60 °C/1 min, followed by final extension at 72 °C/5 min. For each experimental group, qPCR was performed at least twice for every tested gene, and the reactions were always run in triplicate. Following each run of qPCR, a melting curve analysis was performed to validate the presence of a single PCR product.

### 4.11. Statistical Analyses

The data were analyzed using IBM SPSS Statistics 20 and GraphPad Prism 8 software. Two-way repeated measures ANOVA was used to analyze the freezing behavior across the FC and 4 days of FE, with Holm–Sidak correction applied in the post hoc analysis. In addition, we calculated several behavioral parameters, which were analyzed using an independent samples *t*-test. Independent samples *t*-tests were also used to assess ketamine’s effects on c-Fos immunoreactivity, the number of c-Fos + cells across the six ROIs and the protein and gene expression within the two analyzed tissues. All the data are presented as the mean value ± S.E.M., and statistical significance was accepted at *p* < 0.05.

## 5. Conclusions

Our research shows that ketamine improves FE in adolescent males, likely affecting the consolidation and/or recall of extinction memory, with the left IL vmPFC and vHPC as the proposed neural correlates of ketamine’s effects. At the molecular level, we identified Akt-mTOR-GluA1 signaling in the HPC as the key pathway associated with ketamine’s impact on adolescent FE, highlighting it as a promising target for pharmacological augmentation of this process. While our study is the first to comprehensively explore the underlying mechanisms of ketamine’s effects in a fear-related learning paradigm among adolescents, further research is necessary for a better understanding and optimization of ketamine’s potential clinical application for treating fear-related disorders in this vulnerable group.

## Figures and Tables

**Table 1:** Dilutions and catalog numbers of primary antibodies used for Western blot analysis.

Protein	Dilution	Company	Cat. No.
GluA1	1:500	Santa Cruz Biotechnology, Dallas, TX, USA	sc-55509
GluN2A	1:3000	Millipore, Burlington, MA, USA	#07-632
GluN2B	1:1000	Millipore	MAB5782
pErk1/2 (Thr202/Tyr204)	1:1000	Cell Signaling, Danvers, MA, USA	#9101
Erk1/2	1:1000	Cell Signaling	#9102
pmTOR (Ser 2448)	1:1000	Cell Signaling	#2971
mTOR	1:500	Santa Cruz Biotechnology	sc-517464
pAkt (Ser 473)	1:1000	Santa Cruz Biotechnology	sc-7985-R
Akt	1:1000	Cell Signaling	#9272S
BDNF	1:2000	Abcam, Cambridge, UK	ab108319
β-Actin	1:4000	Abcam	ab8227

Abbreviations—GluA1: AMPA receptor subunit 1; GluN2A: NMDA receptor subunit 2A; GluN2B: NMDA receptor subunit 2B; pErk1/2: phosphorylated extracellular signal-regulated kinase 1/2; Erk1/2: extracellular signal-regulated kinase 1/2; pmTOR: phosphorylated mammalian target of rapamycin; mTOR: mammalian target of rapamycin; BDNF: brain-derived neurotrophic factor.

**Table 2:** Nucleotide sequences of specific primers used for qRT-PCR.

Gene	Forward Primer	Reverse Primer
HPRT	5′-TCC TCC TCA GAC CGC TTT T-3′	5′-CCT GGT TCA TCA TCG CTA ATC-3′
BDNF exon IV	5′-CAG AGC AGC TGC CTT GAT GTT-3′	5′-GCC TTG TCC GTG GAC GTT TA-3′
BDNF exon VI	5′-ACA ATG TGA CTC CAC TGC CGG-3′	5′-CGC CTT CAT GCA ACC GAA GTA T-3′
BDNF exon IX	5′-TGC AGG GGC ATA GAC AAA AG-3′	5′-TGA ATC GCC AGC CAA TTC TC-3′

Abbreviations—HPRT: hypoxanthine phosphoribosyltransferase 1; BDNF: brain-derived neurotrophic factor.

## Data Availability

The data presented in this study are contained within the article and Supplementary Materials.

## Supplementary Materials

The following supporting information can be downloaded at https://www.mdpi.com/article/10.3390/ph17060669/s1. Figure S1: Original (unprocessed) images of representative blots.
