# Supplementary figures and images for: Ketamine’s Amelioration of Fear Extinction in Adolescent Male Mice Is Associated with the Activation of the Hippocampal Akt-mTOR-GluA1 Pathway
Source: Pharmaceuticals (Basel). 2024 May 22;17(6):669. doi: 10.3390/ph17060669 (PMC11206546; doi:10.3390/ph17060669)

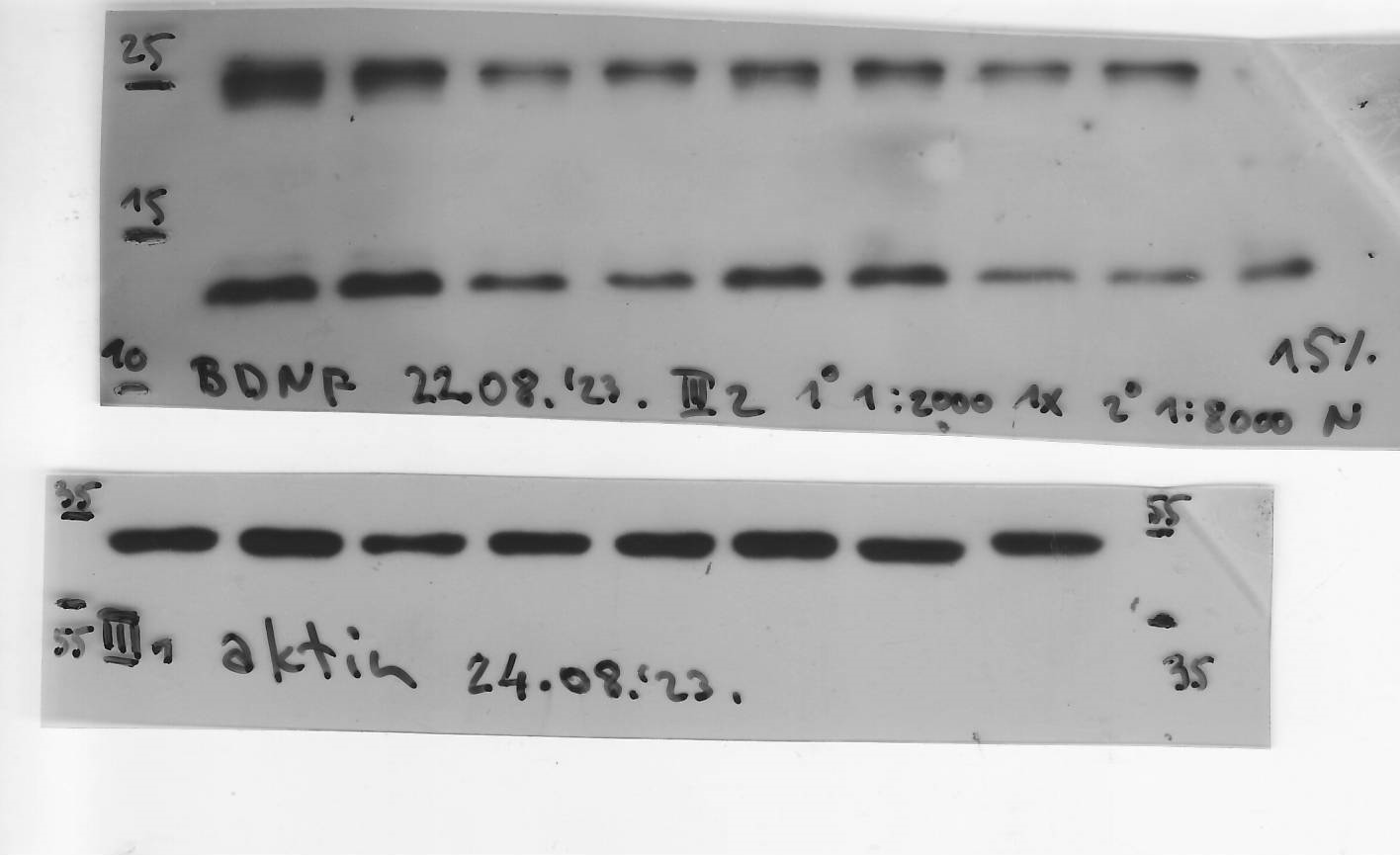

Supplement: Supplementary file 1 [file pharmaceuticals-17-00669-s001.zip › BDNF and Actin.tif]

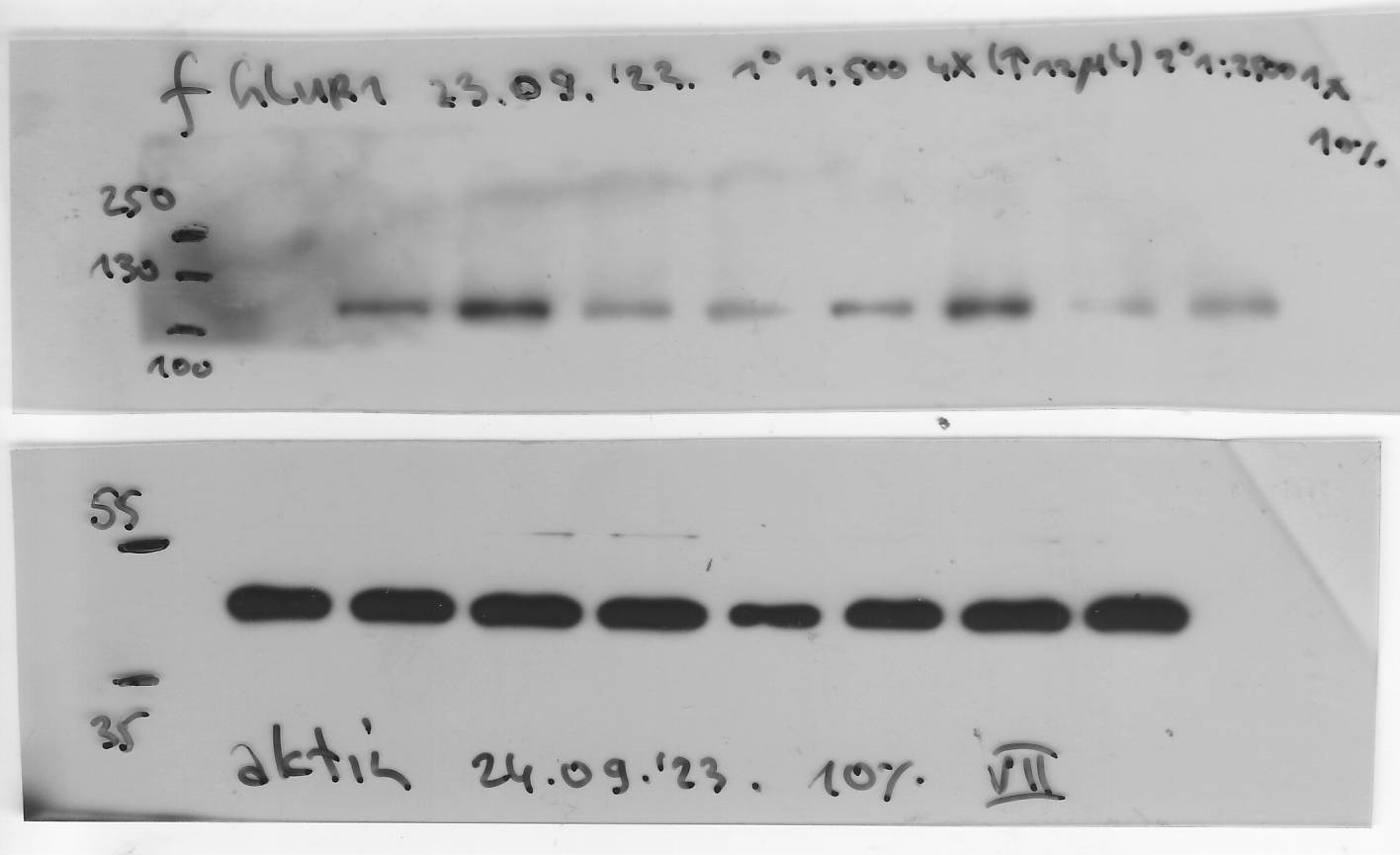

Supplement: Supplementary file 1 [file pharmaceuticals-17-00669-s001.zip › GluA1 and Actin.tif]

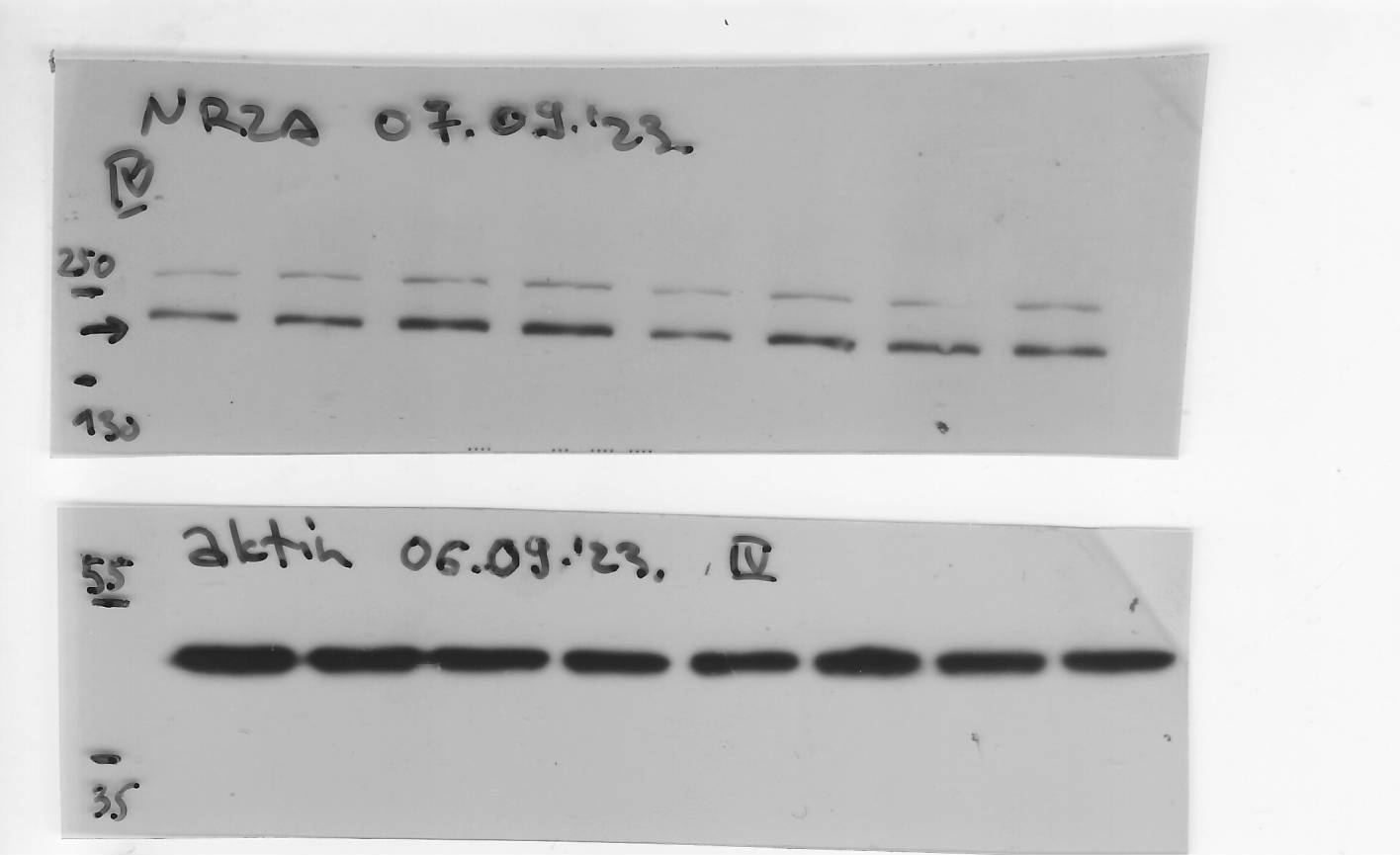

Supplement: Supplementary file 1 [file pharmaceuticals-17-00669-s001.zip › GluN2A and actin.tif]

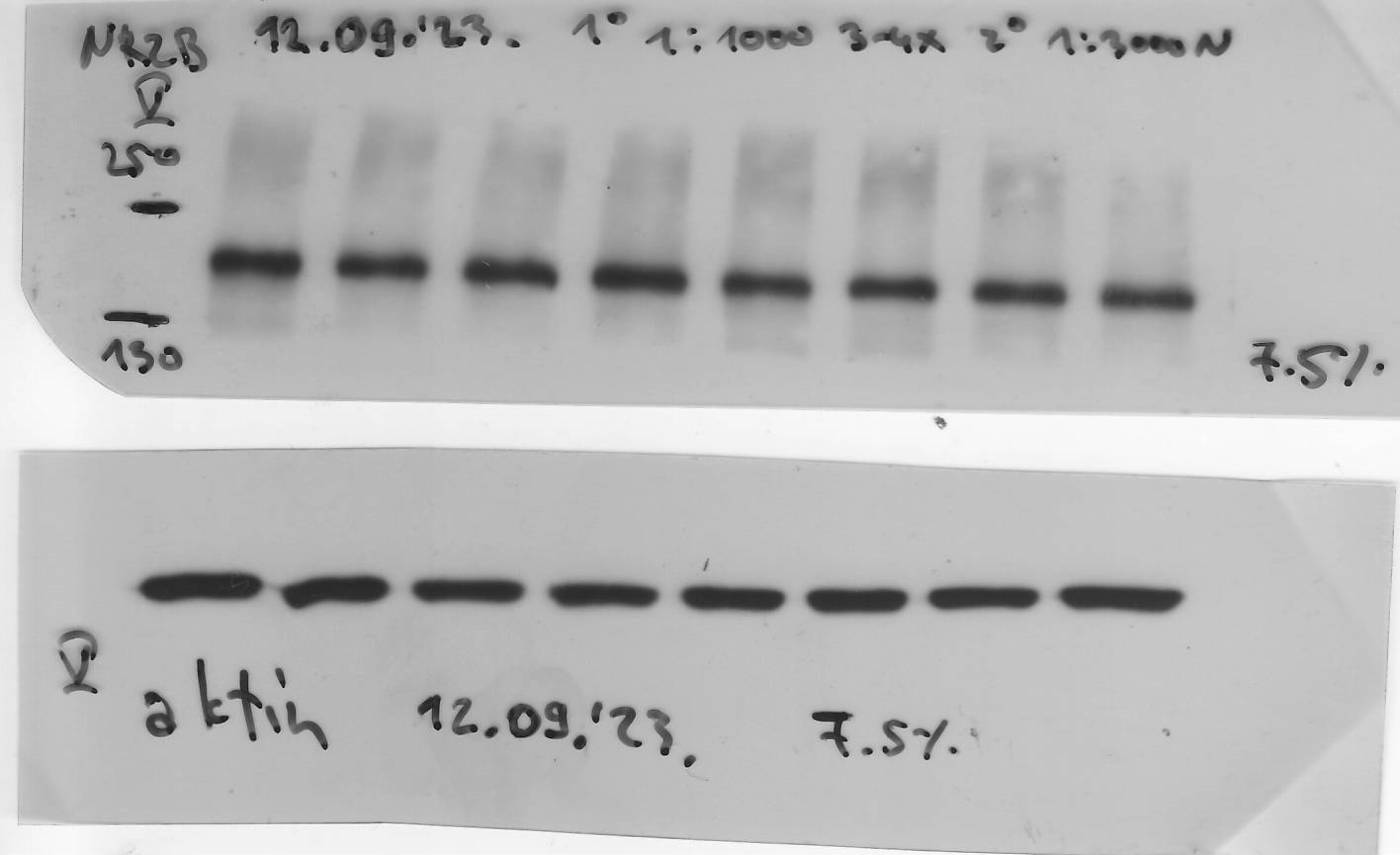

Supplement: Supplementary file 1 [file pharmaceuticals-17-00669-s001.zip › GluN2B and actin.tif]

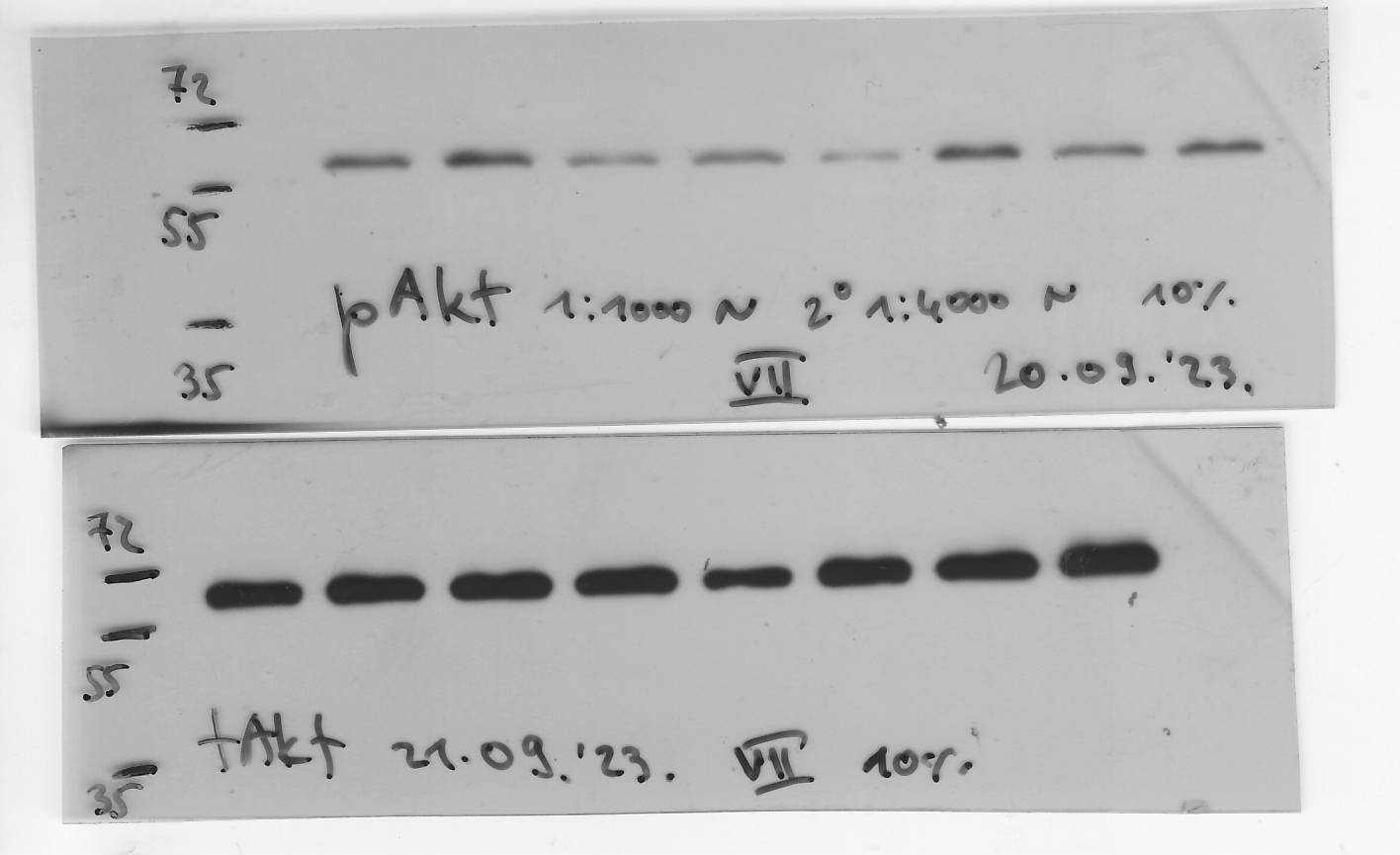

Supplement: Supplementary file 1 [file pharmaceuticals-17-00669-s001.zip › pAkt and tAkt.tif]

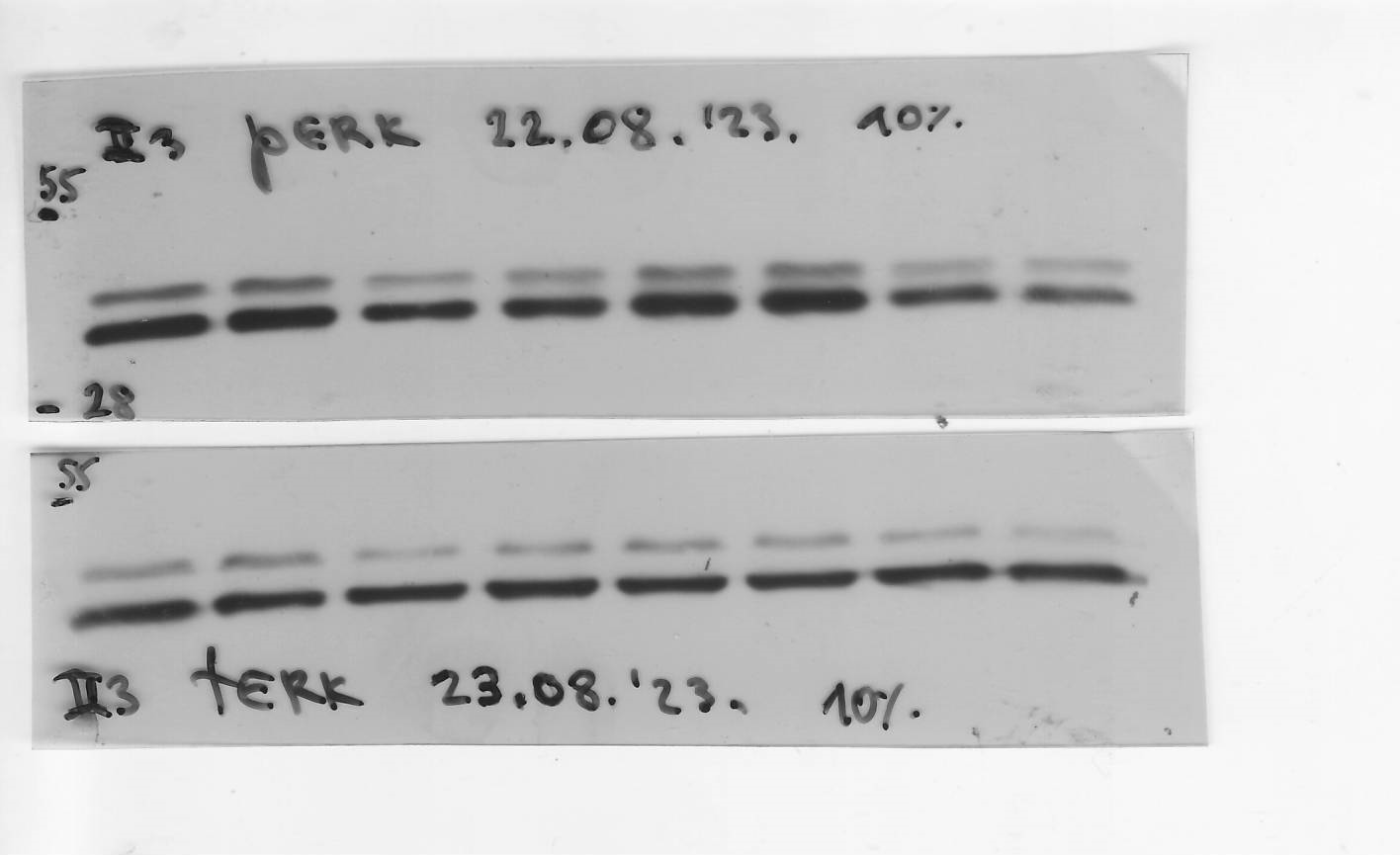

Supplement: Supplementary file 1 [file pharmaceuticals-17-00669-s001.zip › pErk and tErk.tif]

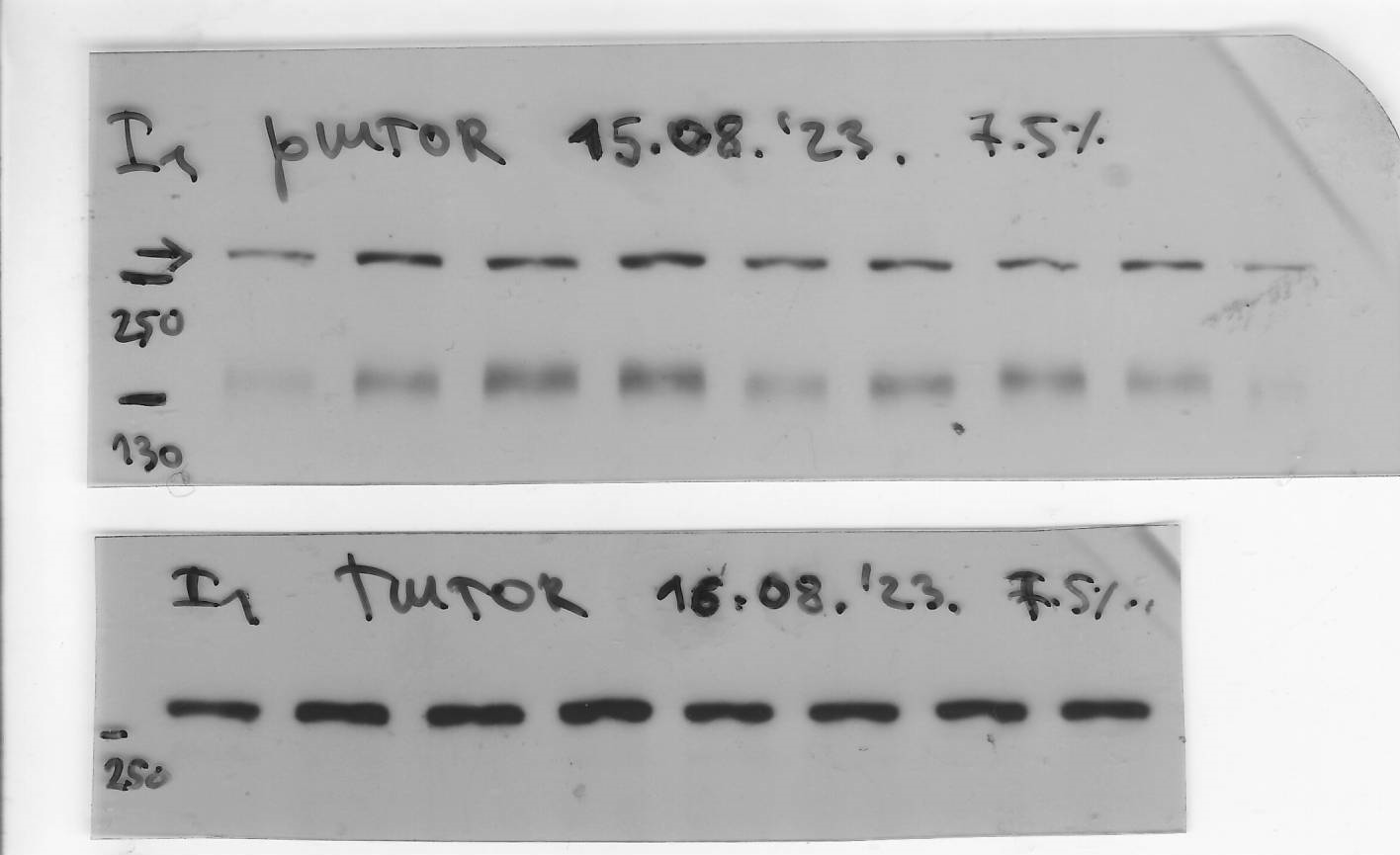

Supplement: Supplementary file 1 [file pharmaceuticals-17-00669-s001.zip › pmTOR and tmTOR.tif]

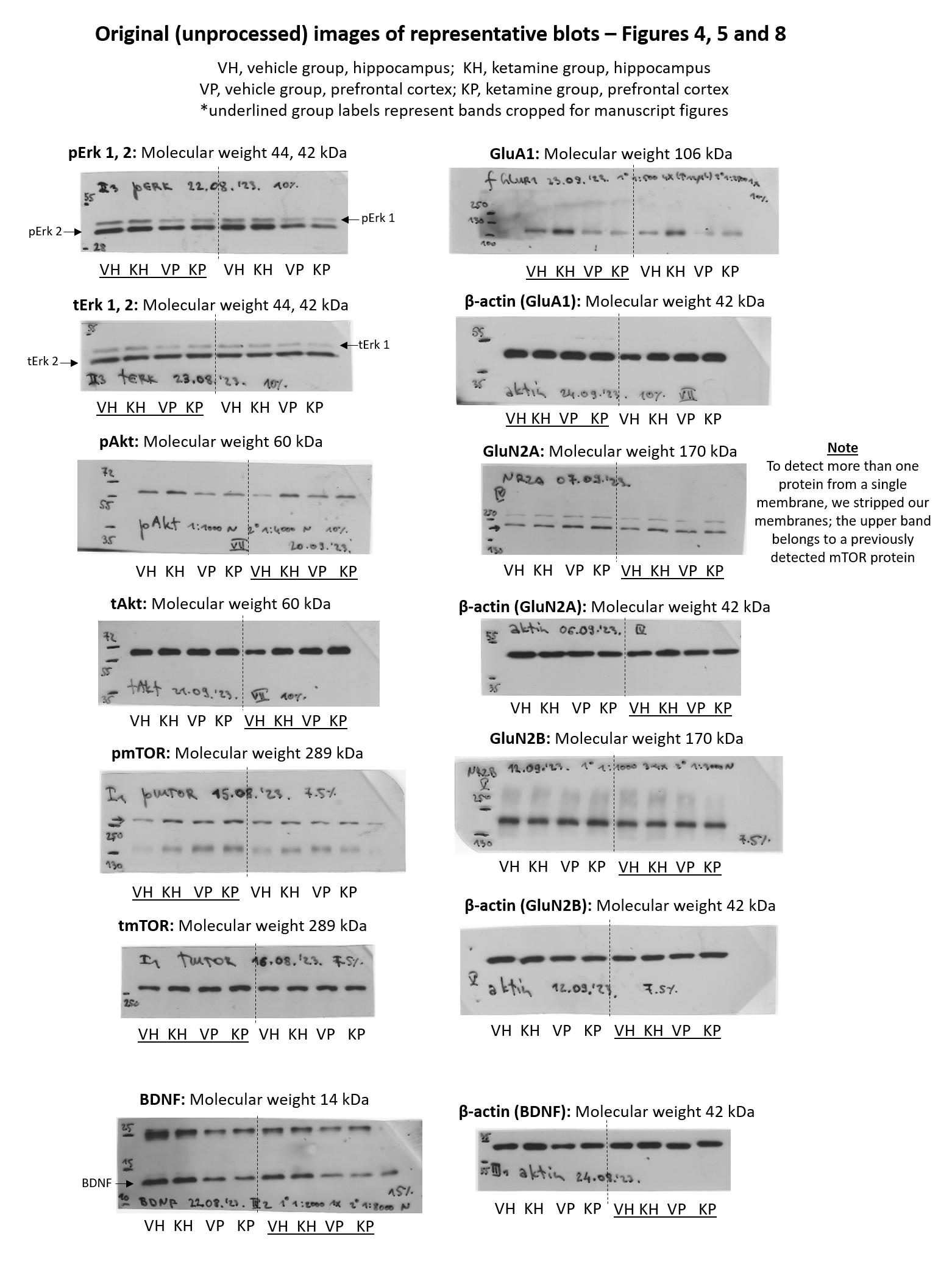

Supplement: Supplementary file 1 [file pharmaceuticals-17-00669-s001.zip › SI Figure 1 1200 dpi.tif]
